# Solar Hydrogen Evolution Boosted by Cu and Pt Single-Atom Sites Anchored on g‑C_3_N_4_ via Magnetron Sputtering Deposition

**DOI:** 10.1021/acsnanoscienceau.6c00011

**Published:** 2026-05-25

**Authors:** Niqab Khan, Erick Jo Prada, Mohammed A. M. Bajiri, Washington Santa Rosa, Flavio L. Souza, Gazi N. Aliev, Wolfgang Theis, Heberton Wender, Valmor R. Mastelaro, Jesum Alves Fernandes, Renato V. Gonçalves

**Affiliations:** † São Carlos Institute of Physics, University of São Paulo, São Carlos 13560-970, São Paulo, Brazil; ‡ Institute of Solid State Physics, Federal University of Bahia, Salvador 40.210-340, Bahia, Brazil; § Brazilian Nanotechnology National Laboratory (LNNANO), Brazilian Center for Research in Energy and Materials (CNPEM), Campinas 13083-970, Brazil and Center for Natural and Human Sciences, 74362 Federal University of ABC (UFABC), Santo André 09210-580, Brazil; ∥ School of Physics & Astronomy, 1724 University of Birmingham, Birmingham B15 2TT, U.K.; ⊥ Laboratory of Advanced Technologies in Energy and Sustainability—LATES, Nano & Photon Research Group, Institute of Physics, Federal University of Mato Grosso do Sul, Campo Grande 79070-900, Brazil; # School of Chemistry, 6123 University of Nottingham, Nottingham NG7 2RD, U.K.

**Keywords:** photocatalytic H_2_ evolution, g-C_3_N_4_, Cu and Pt single atoms, magnetron sputtering, photocatalysis, single-atom active sites

## Abstract

Photocatalytic hydrogen (H_2_) evolution offers a promising solution to environmental pollution and the global energy crisis. Among different photocatalysts, graphitic carbon nitride (g-C_3_N_4_), most known as melon in the literature, is distinguished by its availability, large surface area, low cost, and unique optical and electrical properties. However, the efficiency of pristine g-C_3_N_4_ is limited by rapid electron–hole recombination, presence of charged trapped states and high charge transference resistance. To overcome these challenges, we used a facile magnetron sputtering technique to load Cu and Pt single atoms onto g-C_3_N_4_, confirmed by AC-STEM, XPS, ICP-OES, and XAS characterizations. This approach not only overcomes the problems related to the charge carrier dynamics of the pristine graphitic carbon nitride but also ensures uniform, contamination-free deposition and high distribution of single atoms, thereby optimizing photocatalytic performance. Under solar irradiation (AM 1.5G) for 5 h, the Cu and Pt-loaded g-C_3_N_4_ demonstrated significantly improved photocatalytic activity, achieving H_2_ accumulated values of 93 μmol and 173 μmol, respectively, compared to only 0.3 μmol for pristine g-C_3_N_4_. For comparison, Pt and Cu nanoparticles (NPs)- loaded g-C_3_N_4_ samples were also prepared, achieving H_2_ accumulation values of 86.3 and 24.3 μmol, respectively, compared to pristine g-C_3_N_4_. However, these values are lower than those of Pt and Cu single-atom-loaded samples. The enhanced H_2_ evolution performance is attributed to the deposition of metal single atoms acting as electron traps and active catalytic sites, thus improving electron–hole separation. These findings highlight the potential of sputter depositing single-atom to overcome the inherent limitations of g-C_3_N_4_, paving the way for more efficient and scalable hydrogen production systems.

## Graphical abstract

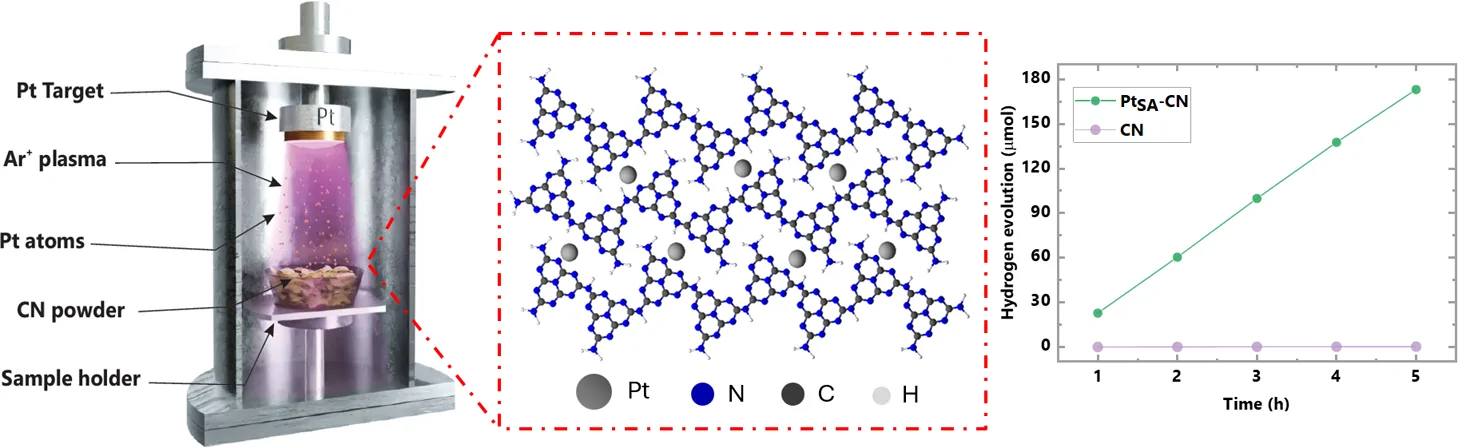

## 1 Introduction

Environmental and energy challenges are among the most pressing issues for sustainable development. Solar energy is a key renewable resource, and photocatalysis has emerged as a promising strategy for converting solar energy into hydrogen in a clean and sustainable manner.^1^ Achieving efficient solar-to-hydrogen conversion requires semiconductor photocatalysts that combine low cost, suitable bandgap energies for visible-light absorption, and long-term chemical and structural stability.^2^ While several photocatalysts, including metal oxides like TiO_2_ and SrTiO_3_, have been extensively explored for photocatalytic hydrogen production, their large bandgap energies (∼3.2 eV) limit their effectiveness under visible light.^3,4^ Recently, attention has been addressed to g-C_3_N_4_, which is regarded as a promising material for solar energy conversion due to its moderate band gap of 2.7 eV (corresponding to an optical wavelength of ∼460 nm), low cost, ease of preparation, and high chemical and physical stability.^5^ However, the practical application of bare g-C_3_N_4_ remains limited due to its high charge recombination rate and electron transfer dynamics, which result in lower photocatalytic activity.^6^

Metal single-atom deposition has emerged as a powerful strategy to enhance photocatalytic performance of several semiconductors photocatalyst. The incorporation of isolated metal atoms into g-C_3_N_4_ has been shown to markedly improve photocatalytic hydrogen evolution by promoting efficient charge separation and suppressing electron–hole recombination.^7–9^ A range of metals, including Ag,^10^ Au,^11^ Pt,^12^ Cu^13^ and Pd,^14^ have been successfully employed to enhance the photocatalytic activity of g-C_3_N_4_, where their high work functions enable effective trapping of photogenerated electrons and facilitate interfacial charge transfer. Among these, Cu and Pt have shown excellent reproducibility and photocatalytic performance when supported on g-C_3_N_4_, making them strong candidates in single atoms catalysis. In fact, g-C_3_N_4_ offers several advantages for the deposition of single atoms (Cu and Pt), such as the presence of 6-fold nitrogen cavities, which can confine and stabilize metal atoms through strong metal–nitrogen interactions. Additionally, the tri-*s*-triazine rings in g-C_3_N_4_, with their abundant and uniformly ordered N species, provide ample coordination sites for single atoms.^15^

Several deposition processes have been previously reported for single-atom deposition on g-C_3_N_4_, including the wet impregnation method, electrostatic absorption, laser ablation, molten salt and reflux method, vapor deposition, and magnetron sputtering deposition.^16–19^ Among these, magnetron sputtering is considered a promising and environmentally friendly technique for depositing nanoparticles on semiconductors. Recent studies have demonstrated the capability of magnetron sputtering to achieve atomic-level dispersion of noble metals on carbon-based supports, such as the successful formation of isolated Pt single atoms on graphene^20^ and the stabilization of Pt single atoms on graphitic carbon nitride (g-C_3_N_4_) obtained from melamine.^21^ These findings confirm that plasma magnetron sputtering enables the controlled formation of single-atom sites without the need for chemical precursors, highlighting its suitability as a versatile and effective technique for fabricating single-atom catalysts for hydrogen evolution reactions.

Herein, we easily synthesized bulk g-C_3_N_4_ via urea pyrolysis at different temperatures and successfully achieved the first deposition of Cu and Pt single atoms on g-C_3_N_4_ using magnetron sputtering. Unlike previous studies, our approach uniquely leverages the uniform nitrogen species in g-C_3_N_4_, which provides abundant coordination sites for single atoms. The photocatalytic activity of Cu and Pt-loaded g-C_3_N_4_ was tested for hydrogen production under solar simulator irradiation, resulting in significantly enhanced photocatalytic activity compared to pristine g-C_3_N_4_. This improvement in H_2_ evolution is attributed to the deposition of Cu and Pt single atoms, which increase electron–hole separation and provide more active sites, thereby boosting photocatalytic performance. The sputtering method represents a significant advancement in the design of efficient single-atom-based photocatalysts for sustainable energy applications.

## 2 Experiments Section

### 2.1 Materials

Urea (Sigma-Aldrich), Cu and Pt targets (Process Material, 99.9%), and Triethanolamine (TEOA, Sigma-Aldrich, 99.5%) were used in this study. Ultrapure water (0.055 μS.cm^–1^) was used for all solution preparations. All other chemical reagents were purchased from Sigma-Aldrich unless otherwise specified.

### 2.2 Synthesis of g-C_3_N_4_

All chemicals used were of reagent grade and were utilized without further purification. Bulk g-C_3_N_4_ was synthesized by pyrolyzing 50 g of urea at 600 °C for 2 h in an alumina crucible with a sealed lid to prevent gas escape. The resulting yellow solid was crushed and kept at 80 °C to prevent agglomeration and remove humidity. The yield was approximately 8% by mass, and the sample was designated as CN.

### 2.3 Sputter Deposition Cu and Pt Single Atoms on g-C_3_N_4_

The deposition of Cu and Pt single atoms on g-C_3_N_4_ was performed using DC-magnetron sputtering. Initially, 200 mg of g-C_3_N_4_ powder was placed into a custom-designed agitator sample holder and subsequently loaded into the magnetron sputtering chamber. The base pressure was approximately 5.0 × 10^–6^ Torr, and then Argon gas (99.99% purity) was introduced to a working pressure of 2 × 10^–2^ Torr. Cu and Pt were deposited for 3 and 10 min (optimized deposition times), respectively, at 15 W power and with a 9 cm target-sample distance. Once the deposition is done, the sample was collected from the chamber, sieved and stored in a vacuum glass desiccator. The samples were designated as Cu_SA_-CN and Pt_SA_-CN.

### 2.4 Deposition of Cu and Pt NPs on g-C_3_N_4_

The Pt deposited g-C_3_N_4_ was synthesized by the wet chemical impregnation method followed by chemical reduction. Initially, the Pt precursor solution was prepared by dissolving 0.5 mg PtCl_2_ in 0.5 M HCl under magnetic stirring and heating at 50 °C to facilitate dissolution. The g-C_3_N_4_ (50 mg) powder was dissolved separately in an ethanol (20 mL) and water (20 mL) mixture (1:1 v/v) and stirred for 1 h. The Pt precursor solution was dropwise added to the g-C_3_N_4_ suspension under continuous stirring. The mixture was stirred at room temperature for 4 h to allow sufficient absorption of Pt species onto the surface of g-C_3_N_4_. The deposited Pt species was reduced by dropping a freshly prepared solution of NaBH_4_ in deionized water. The entire mixture was slowly evaporated at 60 °C, and the final product was dried in an oven at 80 °C overnight. The final sample was named Pt@g-C_3_N_4._ ^22^ The Cu@g-C_3_N_4_ sample was also prepared using the same method.

### 2.5 Photocatalytic Activity

In a typical reaction, 10 mg of catalyst powder was dispersed in 30 mL of solution containing 27 mL denoised water and 3 mL of TEOA (v/v = 90:10), followed by sonication for 15 min. Before the photocatalytic reaction, the suspension was purged with high-purity argon (Ar) to remove dissolved oxygen. The suspension was then placed in an airtight quartz reactor (100 mL), and its pH was measured to be around 9.5. The remaining air was removed by vacuum, and Ar was introduced in sequential and repeated procedures to ensure an oxygen-free environment. The solution was irradiated with an Xe lamp equipped with an AM 1.5G filter, with the lamp intensity adjusted to 500 mW.cm^–2^. The amount of evolved hydrogen (H_2_) was analyzed by gas chromatography (GC, Agilent 7890B) equipped with a thermal conductivity detector (TCD), directly connected to the quartz reactor. High-purity Ar was used as the carrier gas with a flow rate of 1 mL/min. H_2_ evolution was recorded over a total of 5 h. The first measurement was taken in the absence of light, and subsequent measurements were taken hourly, with the evolved H_2_ recorded after automatic injection into the GC.

### 2.6 Sample Characterization

X-ray diffraction (XRD) was used to investigate the structural properties of g-C_3_N_4_ in a Rigaku Ultima IV diffractometer with Cu Kα radiation, 2θ angle ranging from 10° to 80°, and a step scan of 0.02°. Field emission scanning electron microscopy (FESEM) was used to investigate the morphology of the samples in a Zeiss Sigma Gemini operating at an accelerating voltage of 20 kV. UV–vis absorption measurements were performed using a Shimadzu UV-2600 spectrometer fitted with an integrating sphere. Surface composition was analyzed by X-ray photoelectron spectroscopy (XPS) using a Scienta-Omicron ESCA + spectrometer with a monochromatic Al Kα (*hv* = 1486.6 eV) radiation source. The XPS spectra were recorded at a pass energy of 30 eV with 0.05 eV per step. Charging effects were eliminated using a low-energy electron flood gun. Adventitious carbon (C 1s) at 284.8 eV was used to calibrate the XPS spectra. Data analysis was performed using CasaXPS software using a Shirley-type background. X-ray absorption spectroscopy (XAS) measurements were conducted at room temperature at the Cu K-edge and Pt L_3_-edge on the B18 beamline at the Diamond Light Source (SP35043). Extended X-ray Absorption Fine Structure (EXAFS) spectra were obtained for the Cu_SA_-CN, Pt_SA_-CN, Pt and Cu foils, and CuO standards. The energy calibration was performed by aligning the respective absorption edges. Data calibration and normalization were achieved using ATHENA software. Fourier Transform Infrared (FTIR) spectra were recorded over the range of 500 to 4000 cm^–1^ using a Bruker VERTEX 70 FTIR Spectrometer in transmission mode. Aberration Corrected Scanning Transmission Electron Microscopy (AC-STEM) imaging were carried out using JEOL-2100F operated at 200 kV. The microscope was equipped with a Cs probe spherical aberration corrector (CEOS). The probe convergence semiangle was 19 mrad and the collection angle range of the annular dark field (ADF) detectors was set from 72 to 164 mrad. STEM images were captured with an electron probe current 10 pA and a pixel dwell time of 38 μs with a 1024 × 1024-pixel scanning area. The metal Pt and Cu contents were quantified using inductively coupled plasma optical emission spectroscopy (ICP-OES) on a PerkinElmer Optima 2000 spectrometer. Initially, for the sample preparation, around 10 mg of catalyst powder was subjected to microwave-assisted acid digestion in 2 mL of freshly prepared aqua regia (HCl: HNO_3_ 3:1 v/v). The digestion was conducted at 150 °C for 1 h to ensure complete dissolution of both metals and g-C_3_N_4_ components. After the solution had cooled to room temperature, the digested samples were diluted to a final volume of 10 mL using a 5% (v/v) hydrochloric acid solution to stabilize the metal ions before analysis. Brunner-Emmett-Teller (BET) analysis was used to measure the surface area of the pristine sample using Micromeritics Tristar 3000 with hydrogen absorption at 77 K. Photoluminescence (PL) emission spectra were acquired using a Horiba Fluorolog spectrofluorometer operating in front-face (FF) geometry at an incidence angle of 45°. The samples were excited at 350 nm using a monochromator equipped with a 1200 grooves mm^–1^ grating (blaze at 330 nm). Emission spectra were recorded in the range of 365–650 nm with a step size of 1 nm. Both excitation and emission slit widths were set to 2 nm to avoid detector saturation and ensure adequate spectral resolution. A long-pass optical filter (cutoff at 400 nm) was placed in the emission path to suppress scattered excitation light and higher-order diffraction artifacts. The emission signal was detected using a photomultiplier tube (PMT), and all spectra were normalized by the reference detector signal (S1/R1) to correct for fluctuations in the excitation source intensity. The integration time was fixed at 0.08 s, and all measurements were performed under identical instrumental conditions to enable direct comparison among samples.

## 3 Result and Discussion

Bulk g-C_3_N_4_ was synthesized by conventional pyrolysis of urea at 600 °C for 2 h. A yellowish powder was obtained, exhibiting bulk sheet-like morphologies with smooth surfaces, as observed by FESEM (Figure S1) and a BET surface area of 77.0 m^2^ g^–1^ (Figure S2). X-ray diffraction (XRD) patterns of pristine g-C_3_N_4_ (CN) and metal single-atom modified samples (Cu_SA_-CN and Pt_SA_-CN) are shown in Figure 1a. All samples exhibit two characteristic diffraction features of graphitic carbon nitride. The weak peak centered at 12.9° (100) is assigned to the in-plane structural packing of the tri-*s*-triazine (heptazine) units, while the intense and broad peak at 27.7° (002) corresponds to the interlayer stacking of conjugated aromatic CN layers.^23^ Both diffraction peaks are consistent with melon-graphitic carbon nitride. Notably, the broad nature of the diffraction peaks, particularly the (002) reflection, indicates a low degree of long-range order, which is commonly associated with small coherent domain sizes and structural defects such as terminal -NH*x* groups and incomplete polycondensation, associated with a poorly crystalline or semicrystalline g-C_3_N_4_ structure. The deposition of Cu and Pt single atoms by Magnetron sputtering does not lead to the appearance of additional diffraction peaks related to crystalline metal or metal oxide phases, suggesting that the metal species are either atomically dispersed or present in amounts below the detection limit of XRD analysis. To clarify this, metal loading in Cu_SA_-CN and Pt_SA_-CN samples was quantitatively determined by ICP-OES, giving loadings of 0.5 and 0.8 wt % respectively. Furthermore, the preservation of the CN diffraction features indicates that the fundamental g-C_3_N_4_ framework remains intact after metal deposition.

**Figure 1:**
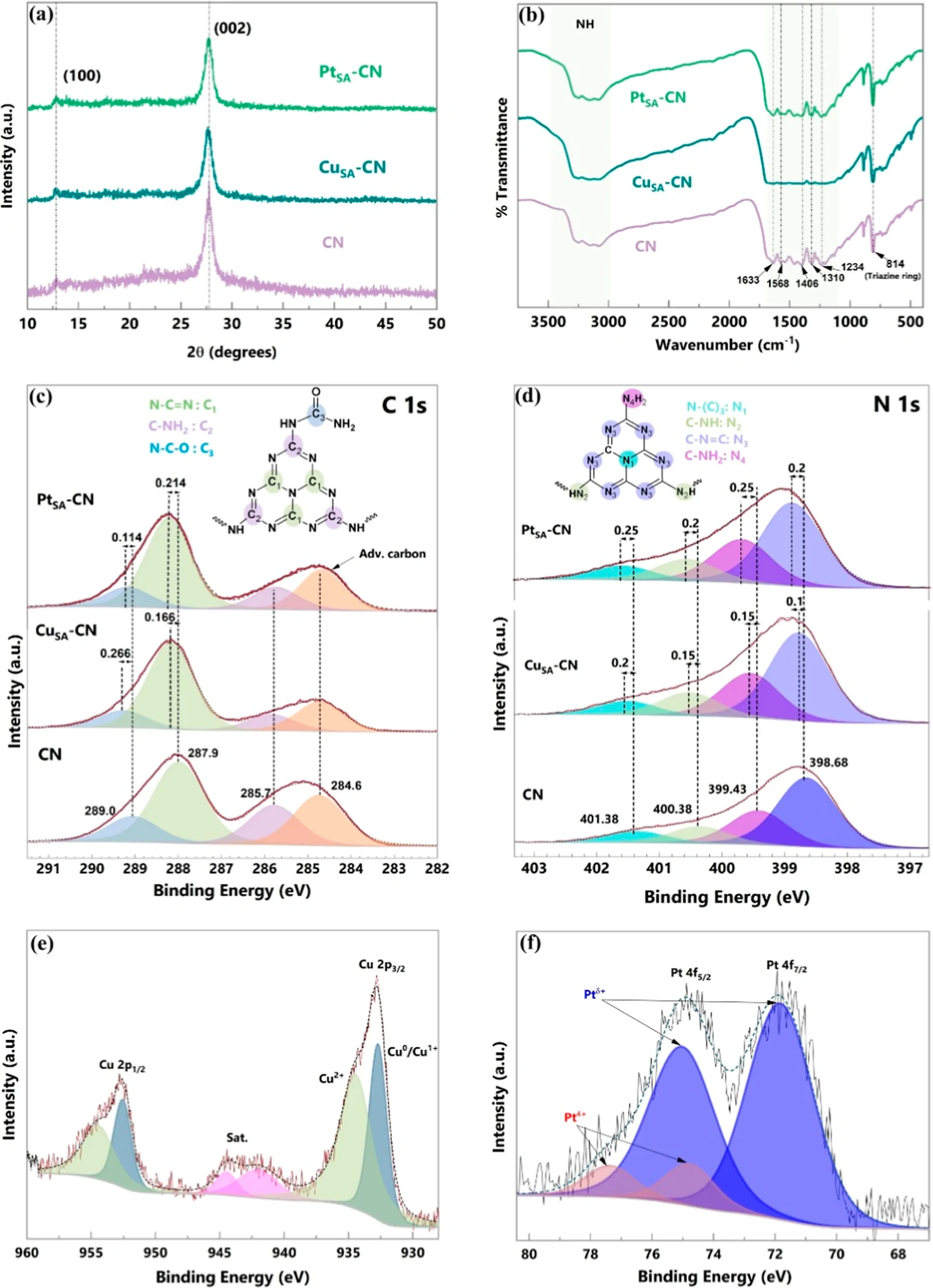
(a) XRD pattern and (b) FTIR spectrum of bulk CN, Cu_SA_-CN and Pt_SA_-CN. High-resolution XPS in the (c) C 1s, (d) N 1s, (e) Cu 2p, and (f) Pt 4f regions. Spectra (e) and (f) refer to the Cu_SA_-CN and Pt_SA_-CN samples, respectively.

FTIR measurements were performed for all three samples, showing a typical spectrum of pristine g-C_3_N_4_ (Figure 1b).^24^ A band at 814 cm^–1^ is clearly visible and corresponds to the breathing mode of the tri-*s*-triazine/heptazine ring, which is widely regarded as a vibration mode for g-C_3_N_4_.^25^ The observation of these features in all samples further confirms the successful formation of the graphitic carbon nitride structure. In the 1200–1700 cm^–1^ region, multiple intense bands are assigned to the stretching modes of C–N and C=N bonds in the heterocyclic network, characteristic of the heptazine (tri-*s*-triazine) units, which serve as crucial building blocks in graphitic carbon nitride.^26^ The persistence of this “fingerprint region” across CN, Cu_SA_-CN and Pt_SA_-CN indicates that the polymeric CN backbone is preserved, and that the deposition of Cu or Pt by Magnetron sputtering does not disrupt the fundamental covalent structure of the carbon nitride framework. Additionally, the spectral features of Pt_SA_-CN remain comparable to pristine CN, which indicates that atomically dispersed Pt induces minimal structural deformation. On the other hand, Cu_SA_-CN exhibits significantly weakened bands, which can be attributed to the formation of Cu subclusters. The formation of subclusters interacts more strongly and nonuniformly with the g-C_3_N_4_ matrix, which leads to structural distortion and partial coverage of active sites, thereby suppressing the vibrational modes.^27^ A broad band between ∼3000–3500 cm^–1^ is observed for all samples and is attributed to N–H stretching vibrations of terminal amine groups (−NH_2_, –NH−) and hydrogen-bonded species, together with possible contributions from O–H stretching of adsorbed water.^28^ This feature is common in g-C_3_N_4_ prepared by thermal condensation because incomplete polycondensation leaves -NH*x* terminations.^29^

X-ray photoelectron spectroscopy (XPS) was performed to further investigate the chemical compositions and corresponding chemical states of the prepared samples. Figure 1c,d show high-resolution C 1s and N 1s spectra for pristine g-C_3_N_4_ (CN) as well as Cu and Pt-loaded g-C_3_N_4_ (Cu_SA_-CN and Pt_SA_-CN). The C 1s spectrum of pristine g-C_3_N_4_ (Figure 1C) can be deconvoluted into four characteristic components. The dominant peak at 287.9 eV is assigned to sp^2^ carbon in N–C = N units of the heptazine (tri-*s*-triazine) rings, representing the fingerprint of the g-C_3_N_4_ framework.^30^ A secondary C 1s component at 285.7 eV is typically assigned to C-NH_*x*_ species located at the edges of heptazine units and/or associated with structural defects in the g-C_3_N_4_ framework.^31^ The higher binding-energy feature at 289.0 eV is attributed to N–C–O or C–O species, originating from adsorbed oxygen-containing groups. In addition, a peak at ∼284.6 eV arises from adventitious carbon (C–C/C = C) from surface hydrocarbon contamination. Altogether, the C 1s profile of CN is dominated by the N–C = N contribution, confirming the preservation of a highly condensed heptazine network with minor contributions from defect-related and oxidized carbon species. The N 1s characteristic peaks for CN sample were observed at binding energies of 398.6, 399.4, 400.3, and 401.3 eV, respectively corresponding to sp^2^-bonded nitrogen (C–N = C) in the triazine rings, amino group (C–NH_2_), C–N–H and quaternary N.^32–35^ The binding energies of C 1s and N 1s for the Cu_SA_-CN sample shifted to higher values as compared to the CN sample. This shift is attributed to surface electron transfer resulting from strong chemical coupling between the metal atoms and the g-C_3_N_4_ support.^36^ A similar shift in binding energies was observed for the Pt_SA_-CN sample, where the C 1s and N 1s energies also increased. This shift is attributed to the loading of Pt atoms, which leads to electron transfer to the single Pt atoms, thereby lowering the nitrogen density on the g-C_3_N_4_ surface.^37^ Importantly, despite these shifts, the persistence of the ∼288.7 eV N–C = N peak in all samples confirms that the heptazine backbone of g-C_3_N_4_ remains structurally intact after metal incorporation.

The Cu 2p spectrum (Figure 1e) exhibits a Cu 2p_3/2_ envelope composed of contributions assigned to Cu^0^/Cu^+^ (∼932 eV) and Cu^2+^ (∼934 eV), together with pronounced shakeup satellites (∼940–945 eV), confirming the presence of Cu^2+^ species. The coexistence of cationic Cu states and satellite features is consistent with Cu stabilized in strongly interacting surface environments on g-C_3_N_4_ (e.g., Cu–N_*x*_ coordination), while the residual Cu^0^/Cu^+^ component suggests that a fraction of Cu may exist in reduced or more aggregated forms.^38–40^ The Pt 4f XPS spectrum in Figure 1f provides strong chemical evidence that Pt is predominantly present on g-C_3_N_4_ as highly dispersed, cationic Pt species, consistent with atomically anchored Pt rather than metallic nanoparticles. The Pt 4f_7/2_ peak appears at 71.8 eV, characteristic of positively charged Pt^δ+^, while the corresponding Pt 4f_5/2_ component is located at 74.8 eV. In addition, the presence of a higher-binding-energy component indicates a fraction of Pt in a more oxidized Pt^4+^ state, which can originate from Pt atoms coordinated to more electronegative local environments, such as defect-rich nitrogen sites or residual surface oxygen species. The overall positive binding-energy shift relative to metallic Pt reflects strong electronic interaction between Pt and the nitrogen-rich g-C_3_N_4_ framework, indicating electron transfer from Pt to coordinated nitrogen atoms.^41^ This behavior is consistent with the stabilization of isolated Pt atoms via Pt–N_*x*_ coordination rather than the formation of Pt clusters or aggregated Pt nanoparticles.^42^

The optical absorption properties of CN and Cu_SA_-CN were investigated using UV–vis spectroscopy. As shown in Figure S3, all samples exhibited absorption peaks within the visible region, corresponding to different absorption edges. These edges allowed for the calculation of the bandgap energies, which were found to be 2.78, 2.85, and 2.76 eV for CN, Pt-loaded (Pt_SA_-CN), and Cu-loaded samples (Cu_SA_-CN). Notably, the Cu_SA_-CN sample had the lowest bandgap energy, which may be attributed to the increased presence of Cu atoms, potentially narrowing the bandgap. Shortly, the incorporation of Cu atoms shifts the visible light absorption threshold to longer wavelengths, which can affect the optical properties of carbon nitride.^43^

The surface morphology and microstructure of the prepared samples were investigated by FESEM and AC-STEM. The FESEM images of the CN revealed bulk sheet-like morphologies with smooth surfaces.^44^ The magnetron sputtering scheme for depositing Cu single atoms on bulk g-C_3_N_4_ is illustrated in Figure 2a, showing the Cu target, Cu atoms, and g-C_3_N_4_ inside the sample holder. Similarly, Figure 2b depicts the scheme for Pt deposition on bulk g-C_3_N_4_, representing the Pt target, Pt atoms, and g-C_3_N_4_ in the sample holder. The DC magnetron sputtering technique, which is traditionally employed for the deposition of thin films on flat substrates, has also shown considerable potential for the deposition of nanoparticles on powder supports.^45–47^ More recently, this approach has been successfully extended to the deposition of single atoms on powder substrates.^48,49^ In this technique, a metallic target is bombarded by highly energetic Ar^+^ ions, leading to the ejection of one or more atoms from the target that subsequently travel toward the substrate.^50^ In the present work, the g-C_3_N_4_-based powder was continuously agitated in the sample holder, enabling uniform exposure of the support to the sputtered Pt and Cu species. This dynamic configuration facilitates the efficient capture of incoming atoms at coordination sites on the g-C_3_N_4_ sheets, promoting their stabilization as isolated single atoms coordinated to nitrogen or carbon sites within the g-C_3_N_4_ framework.

**Figure 2:**
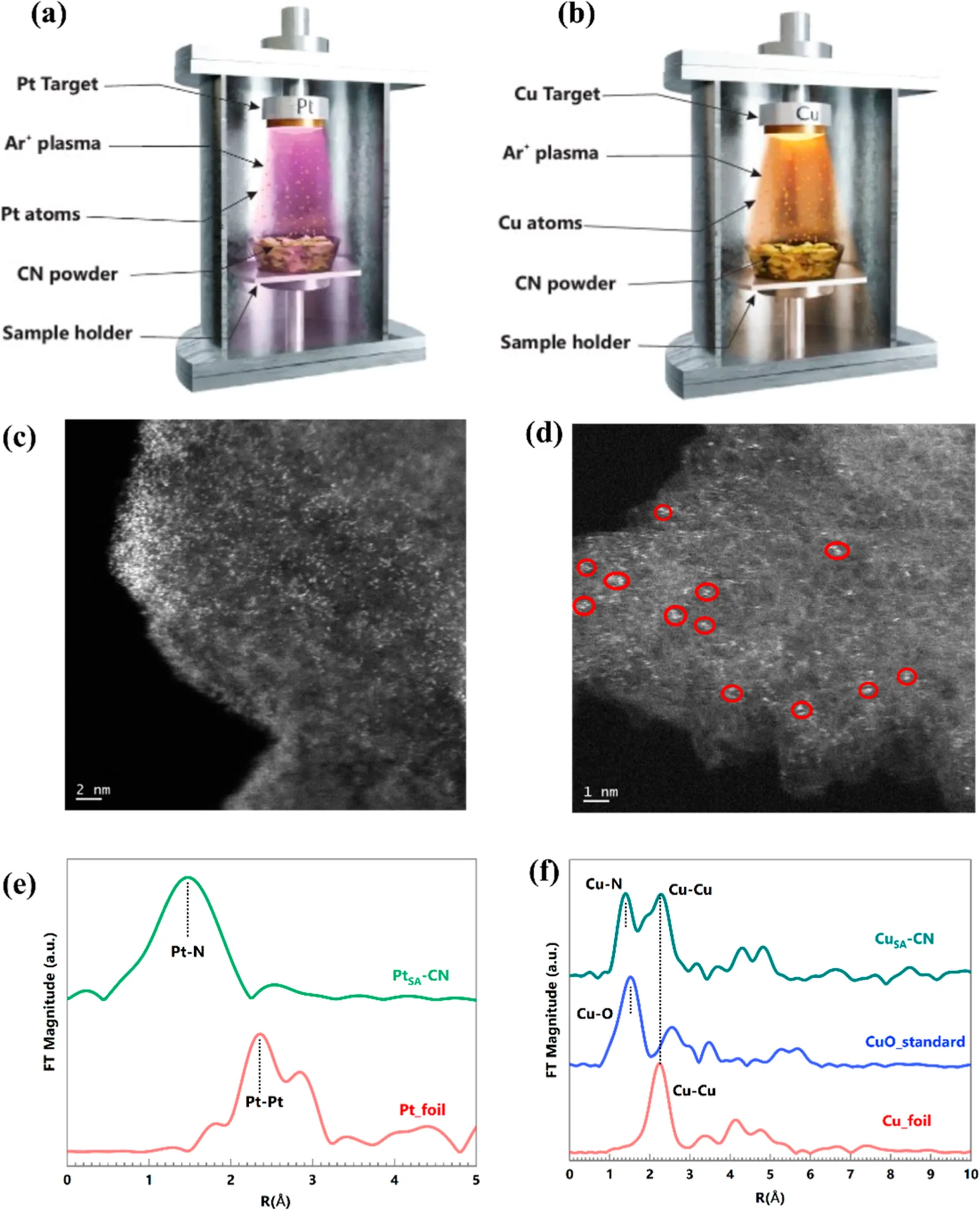
Scheme for single atoms deposition of (a) Pt and (b) Cu via magnetron sputtering. Aberration Corrected Scanning Transmission Electron Microscopy (AC-STEM) of Pt_SA_-CN (c) and Cu_SA_-CN (d) showing homogeneous dispersion Pt and Cu atoms at g-C_3_N_4_ surface as well as formation of Cu subnanocluster, which is highlighted (red circled). Fourier transformed EXAFS spectra of (e) Pt_SA_-CN and Pt foil, (f) Cu_SA_-CN, Pt foil and CuO standard.

Figure 2c shows an AC-STEM image of the Pt single atoms loaded on g-C_3_N_4_ (Pt_SA_-CN), which similarly demonstrates the uniform distribution of Pt atoms on the g-C_3_N_4_ sheets, as seen in the encircled region. Unlike chemical methods, DC-sputtering allows for the direct deposition of single atoms onto the surface of the support material, as observed in the images. In contrast, most chemical methods tend to localize single atoms primarily at the edges or within the lamellae of the structure, such as in g-C_3_N_4_, which can reduce the catalytic potential of the material by obscuring many single-atom sites in less accessible regions. The corresponding AC-STEM images of the Cu-deposited g-C_3_N_4_ (Cu_SA_-CN), shown in Figure 2d, confirm the presence of isolated Cu single atoms on the g-C_3_N_4_ support. Moreover, these images demonstrate a uniform spatial distribution of Cu atoms across the g-C_3_N_4_ matrix. In addition to these single atoms, a few subnanoclusters can also be observed, as highlighted in Figure 2d. This coexistence of single atoms and small clusters suggests partial aggregations of Cu species. TEM images of Pt and Cu nanoparticles (NPs) supported on the g-C_3_N_4_ are also presented in Figure S4, revealing average particle sizes of approximately 10.6 and 7.2 nm, respectively. Unlike the isolated atomic dispersion observed in Cu and Pt single-atom deposited samples, the Cu and Pt nanoparticle deposited samples exhibit clearly distinguishable larger particle sizes. This comparison highlights structural differences between nanoparticle-loaded and single-atom-loaded g-C_3_N_4_, which are expected to significantly influence their electronic properties and catalytic behavior.

The local coordination structures of Pt and Cu deposited on g-C_3_N_4_ were elucidated by X-ray absorption near-edge spectra (XANES) and Fourier transformed EXAFS (FT-EXAFS) analysis, which provides direct insight into the atomic-scale environment of the metal centers. The normalized XANES spectra for the Pt_SA_-CN sample measured at the PtL_3_-edge demonstrate positively charged Pt centers (Figure S5a).^51^ For the same sample, the FT-EXAFS spectrum displays a single dominant peak at ∼1.50 Å, which is assigned to Pt–N scattering from coordination with nitrogen atoms in the g-C_3_N_4_ framework. Importantly, the characteristic Pt–Pt peak at ∼2.5 Å observed in the Pt foil is completely absent, unambiguously demonstrating that Pt atoms are not aggregated but are instead atomically dispersed and stabilized by N coordination sites (Figure 2e).^52^ This structural result is fully consistent with the Pt 4f XPS spectrum, which shows Pt in positively charged Pt^δ+^ states rather than metallic Pt^0^, reflecting strong electronic coupling between Pt atoms and the nitrogen-rich support. Furthermore, to investigate the oxidation state of the Cu single-atom in Cu_SA_-CN sample, X-ray absorption energy near-edge structure (XANES) of Cu foil, CuO, and Cu_SA_-CN were performed as illustrated in Figure S5b. The findings suggested that the XANES spectrum of Cu_SA_-CN falls between the spectra of Cu^0^ and CuO, indicating that the oxidation state of the Cu single-atom was between 0 and +2.^53^ For Cu_SA_-CN, the FT-EXAFS spectrum is dominated by a strong low-R peak at ∼1.4–1.7 Å, attributed to Cu–N coordination, confirming that most Cu atoms are anchored to the g-C_3_N_4_ network through bonding to light scatterers (Figure 2f).^39^ A weaker Cu–Cu contribution is observed at ∼2.2–2.5 Å, indicating that, although Cu is predominantly atomically dispersed, a minor fraction of Cu atoms forms dimers or subnanometric clusters. This interpretation is consistent with the AC-STEM observations, which mainly reveal isolated Cu atoms, while a few subnanoclusters can also be identified (Figure 2d). This interpretation is supported by the Cu 2p XPS spectrum, which shows predominantly cationic Cu species (Cu^2+^) interacting with the g-C_3_N_4_ framework, with only a minor Cu^0^/Cu^+^-like contribution consistent with limited aggregation. Together, the EXAFS results demonstrate that DC-magnetron sputtering enables the stabilization of Pt exclusively as isolated single atoms and Cu predominantly as single atoms with minor Cu–Cu pairing, anchored by strong metal–nitrogen coordination on g-C_3_N_4_.

The photocatalytic activity of pure and single-atom-loaded g-C_3_N_4_ (Cu or Pt) was tested for hydrogen evolution under visible light irradiation for 5 h in the presence of TEOA as a sacrificial agent. Upon exposure of the prepared Pt and Cu loaded g-C_3_N_4_ to light irradiation, electrons from the valence band are excited to the conduction band of g-C_3_N_4_ and are subsequently transferred to the isolated Pt or Cu atoms. These isolated atoms serve as active centers for H_2_ evolution. On the other hand, holes remaining in the valence band are efficiently consumed by the triethanolamine (TEOA), which suppresses charge recombination and enhances the photocatalytic efficiency. Notably, pristine CN exhibited minimal photocatalytic activity, generating only 0.4 μmol of H_2._ The lower photocatalytic response of the CN sample is attributed to its rich surface defects, which lead to electron–hole (e^–^/h^+^) recombination.^54^ The photocatalytic H_2_ evolution of g-C_3_N_4_ (CN) loaded with Pt and Cu single atoms is also illustrated in Figure 3. The Pt single atom loaded on g-C_3_N_4_ for 5 min resulted in higher photocatalytic activity, producing 74.2 μmol (Figure 3a). Similarly, the Pt loaded on g-C_3_N_4_ for 10 min (Pt_SA_-CN-10 min) demonstrated an even greater photocatalytic hydrogen evolution of 173 μmol, representing a 432-fold increase compared to pristine CN. Increasing the deposition time of the Pt loading on g-C_3_N_4_ for 15 min (Pt_SA_-CN-15 min) resulted in 124.8 μmol. Similarly, the Cu-loaded on g-C_3_N_4_ for 1 min (Cu_SA_-CN-1 min) resulted in 1.2 μmol of H_2_. Interestingly, increasing the deposition time of Cu loading on g-C_3_N_4_ for 3 min (Cu_SA_-CN-3 min) exhibited a substantially higher photocatalytic hydrogen evolution of 93 μmol, representing a 232-fold increase compared to pristine CN (Figure 3c). However, exceeding the amount of Cu loading on g-C_3_N_4_ resulted in lower H_2_ production; the Cu-loaded g-C_3_N_4_ for 5 min (Cu_SA_-CN-5 min), resulting in 18.7 μmol of H_2_. The enhancement in photocatalytic H_2_ evolution of g-C_3_N_4_ is attributed to the presence of Cu and Pt single atoms, which play a crucial role in promoting efficient electron–hole separation.^55,56^ The electron–hole separation is further confirmed by PL analysis, where the Pt and Cu single atoms deposited on g-C_3_N_4_ sample exhibits a significant quenching of the PL intensity (Figure S6). Furthermore, to enable a direct comparison, Pt and Cu nanoparticles were also prepared using conventional chemical methods and loaded onto g-C_3_N_4_ at the same metal contents as the optimized samples (0.8 wt % for Pt@g-C_3_N_4_ and 0.5 wt % for Cu@g-C_3_N_4_). The photocatalytic hydrogen evolution activities of these samples are presented in Figure 3b,d, where the Pt@g-C_3_N_4_ exhibits higher photocatalytic activity than the Cu@g-C_3_N_4_ sample, producing 86.3 and 24.3 μmol of H_2_, respectively, under identical experimental conditions. However, both values are lower than those obtained for the Pt_SA_-CN and Cu_SA_-CN samples. The lower activity of these samples clearly demonstrates the advantage of the metal single-atom cocatalysts in achieving high activity while maximizing atom utilization. The higher photocatalytic hydrogen evolution (HER) activity observed for Pt_SA_-CN compared to Cu_SA_-CN is attributed to the nature of the Pt species and their interaction with the g-C_3_N_4_ support. In the Pt-loaded sample, Pt is predominantly stabilized as atomically dispersed single sites, which maximize metal utilization and provide well-defined active centers for HER. Moreover, these Pt single atoms promote efficient charge separation by acting as electron trapping sites through strong coordination with the nitrogen framework of g-C_3_N_4_, thereby suppressing electron–hole recombination.^58^ In contrast, although Cu is also present as single-atom species, its comparatively lower intrinsic activity and less favorable electronic interaction with the g-C_3_N_4_ matrix lead to reduced photocatalytic efficiency. Furthermore, Pt is present as atomically dispersed single sites, whereas Cu-loaded g-C_3_N_4_ exhibits both single atoms and subnanoclusters, as confirmed by AC-STEM and XAFS (Figure 2). Thus, the isolated Pt sites provide uniform active centers and strong metal support interactions, leading to enhanced charge separation and superior HER activity, while Cu subnanoclusters reduce atomic utilization and overall catalytic efficiency.^57^

**Figure 3:**
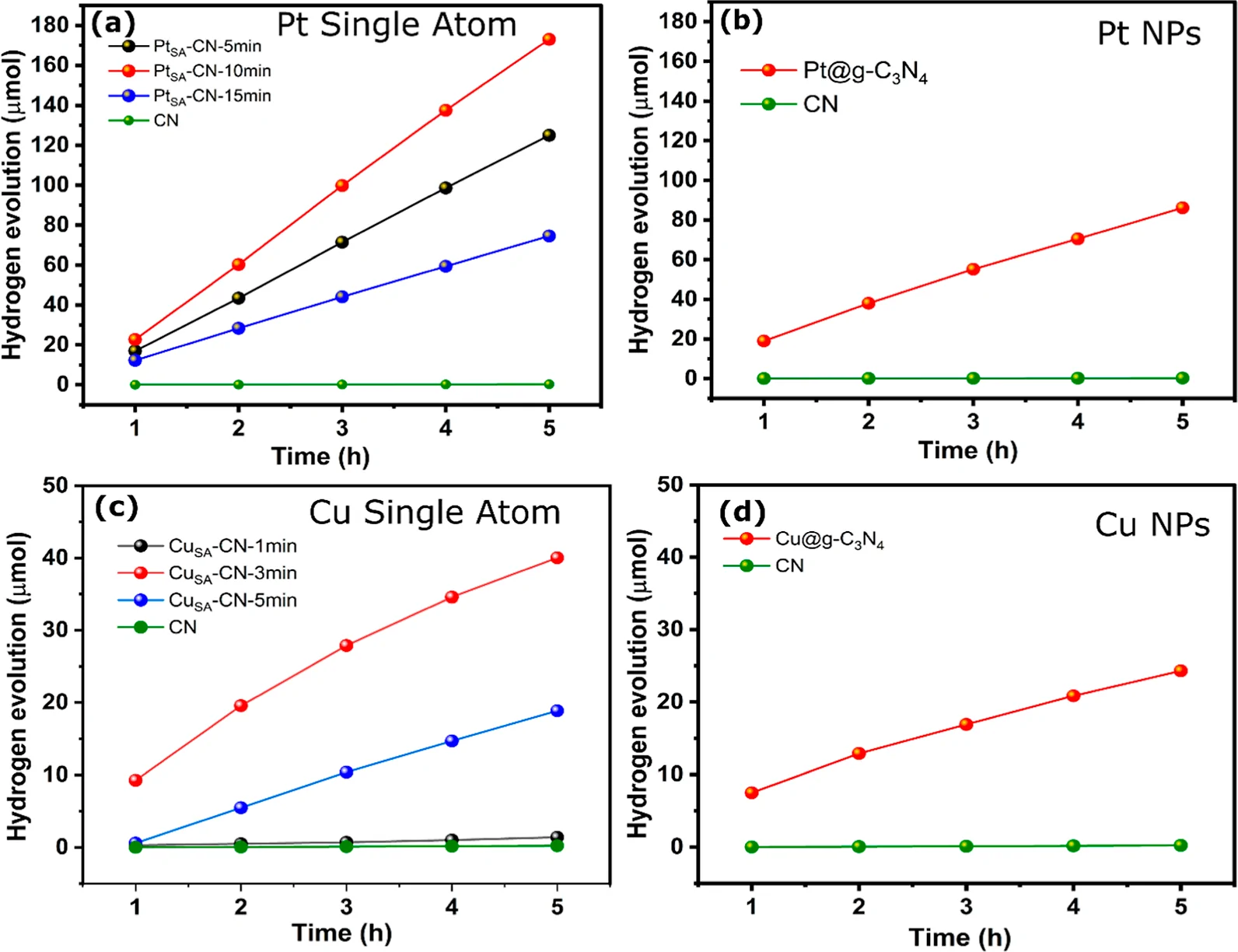
Photocatalytic hydrogen evolution of g-C_3_N_4_ loaded with (a) different deposition time of Pt single atoms, (b) Pt nanoparticles (NPs, 0.8 wt %), (c) different deposition time of Cu single atoms, and (d) Cu nanoparticles (NPs, 0.5 wt %). Photocatalytic hydrogen evolution experiments were carried out using 10 mg of photocatalyst dispersed in 30 mL of an aqueous solution containing 10 vol % triethanolamine (TEOA). The system was irradiated under simulated solar light (AM 1.5G) with an intensity of 500 mW cm^–2^ at 30 °C for 5 h.

## 4 Conclusion

In summary, melon g-C_3_N_4_ were synthesized by pyrolysis of urea, followed by the deposition of Cu and Pt single atoms using the magnetron sputtering technique. This method ensures uniform coating of Cu and Pt atoms on the surface of g-C_3_N_4_, as confirmed by AC-STEM imaging. The presence of Cu and Pt single atoms was further verified by XPS and XAS analysis. The photocatalytic activity of pure, Cu- and Pt-loaded g-C_3_N_4_ was tested for H_2_ evolution from water with TEOA as a sacrificial agent. The highest H_2_ production of 93 and 173 μmol compared to only 0.3 μmol for the pure g-C_3_N_4_ was achieved with Cu and Pt single-atom loaded samples, respectively. For comparison, Pt and Cu nanoparticles (NPs)- loaded g-C_3_N_4_ samples were also prepared using the chemical impregnation method, achieving H_2_ accumulation values of 86.3 and 24.3 μmol, respectively, compared to pristine g-C_3_N_4_. However, these values are lower than those of Pt and Cu single-atom-loaded samples. The enhancement in photocatalytic H_2_ evolution is attributed to the deposition of Cu and Pt atoms, which accelerate electron transfer from g-C_3_N_4_ to the active sites, promoting efficient electron–hole separation. The electron–hole separation is further confirmed by PL analysis, where the Pt and Cu deposited g-C_3_N_4_ sample exhibits a significant quenching of the PL intensity, elucidating suppression of charge recombination. Additionally, Cu and Pt assist in trapping electrons from the photocatalyst, further improving charge separation and, consequently, boosting photocatalytic H_2_ evolution.

## Supplementary Material



## Notes

FESEM images of bulk g-C_3_N_4_ synthesized by urea pyrolysis; BET surface area analysis of pristine g-C_3_N_4_; XPS binding energy shifts of N 1s and C 1s core levels for CN, Cu_SA_-CN, and Pt_SA_-CN samples; UV–vis absorption spectra and Tauc plots of pristine and metal-loaded g-C_3_N_4_ samples; HRTEM images of Cu and Pt nanoparticles supported on g-C_3_N_4_; normalized XANES spectra at the Pt L3-edge and Cu K-edge; photoluminescence spectra comparing pristine, single-atom, and nanoparticle-modified g-C_3_N_4_ samples; and supplementary discussion related to charge separation and photocatalytic activity enhancement (PDF)
