# Supplementary material for: Solar Hydrogen Evolution Boosted by Cu and Pt Single-Atom Sites Anchored on g‑C3N4 via Magnetron Sputtering Deposition
Source: ACS Nanosci Au. 2026 May 25;6(4):596–605. doi: 10.1021/acsnanoscienceau.6c00011 (PMC13495495; doi:10.1021/acsnanoscienceau.6c00011)
Supplement: Supplementary file 1 [file ng6c00011_si_001.pdf]

Solar Hydrogen Evolution Boosted by Cu and Pt Single-Atom Sites Anchored on g-C_3N_4 via Magnetron Sputtering Deposition

Niqab Khan [a], Erick Jo Prada [a], Mohammed A. M. Bajiri [a], Washington Santa Rosa[a,b], Flavio L. Souza[c], Gazi N. Aliev [d], Wolfgang Theis[d], Heberton Wender[e], Valmor R. Mastelaro[a], Jesum Alves Fernandes[f] and Renato V. Gonçalves [a]*

[a] São Carlos Institute of Physics, University of São Paulo (USP), 13560-970 São Carlos, SP, Brazil.

[b] Institute of Solid-State Physics, Federal University of Bahia, 40.210-340 Salvador, BA, Brazil

[c] Brazilian Nanotechnology National Laboratory (LNNANO), Brazilian Center for Research in Energy and Materials (CNPEM), Campinas 13083-970, Brazil and Center for Natural and Human Sciences, Federal University of ABC (UFABC), 09210-580, Santo André, Brazil.

[d] School of Physics & Astronomy, University of Birmingham, B15 2TT, Birmingham, UK

[e] Laboratory of Advanced Technologies in Energy and Sustainability – LATES, Nano&Photon Research Group, Institute of Physics, Federal University of Mato Grosso do Sul, Campo Grande 79070-900, Brazil

[f] School of Chemistry, University of Nottingham, NG7 2RD, Nottingham, UK.

The morphology of the pristine g-C_3N_4 (CN) was investigated by scanning electron microscopy (SEM), as shown in Figure S1. The material exhibits a typical layered and corrugated structure formed by stacked sheets with irregular folds and porous interspaces. This morphology is characteristic of graphitic carbon nitride synthesized via thermal polymerization of urea and is beneficial for photocatalytic H_2 production.

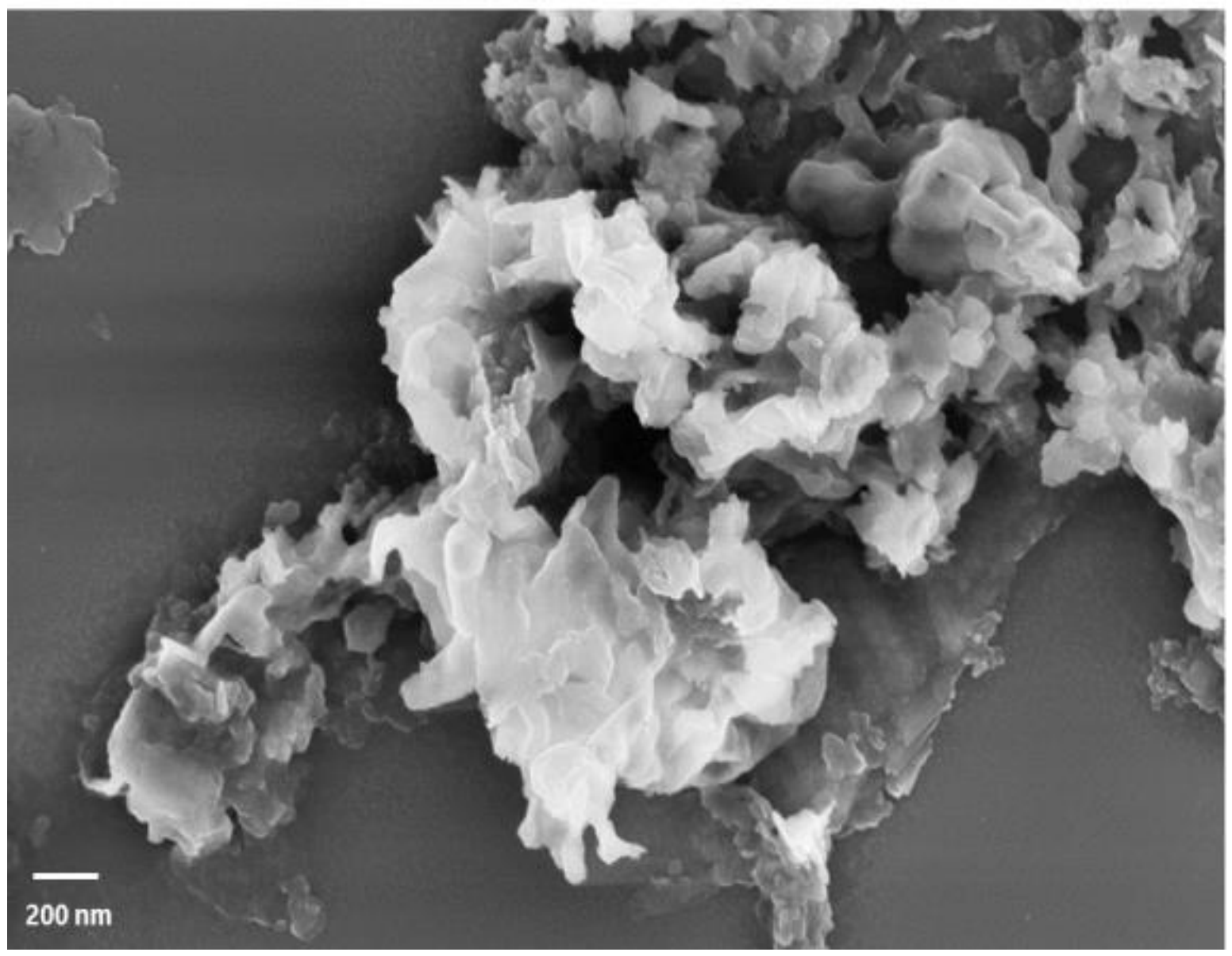

Figure S1. Scanning electron microscopy (SEM) images of bulk g-C_3N_4 (CN) obtained from pyrolysis of urea at 600 C.

The BET surface area analysis for the pristine g-C_3N_4 is illustrated in Figure S2, exhibiting a higher surface area of 77.0 m^2 g^{-1}. [1] The larger surface area offers abundant anchoring sites for cocatalyst deposition, which facilitates uniform dispersion of metal species. The uniform dispersion of metal species is crucial for maximizing active sites and enhancing photocatalytic H_2 performance.

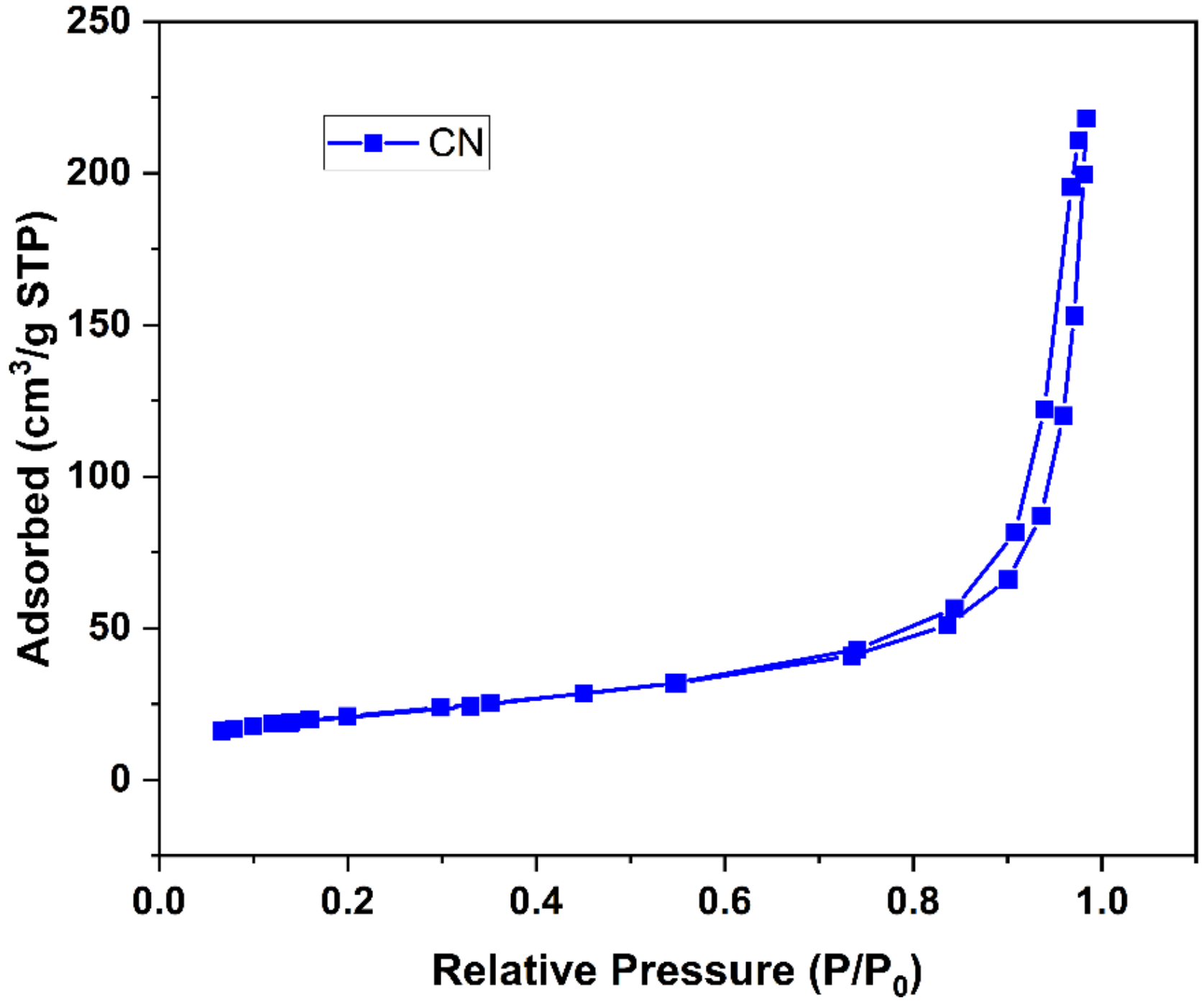

Figure S2. N_2 adsorption-desorption isotherm of pristine g-C_3N_4 obtained from Brunauer-Emmett-Teller (BET) analysis, showing a specific surface area of 77.0 m^2 g^{-1} and characteristics associated with mesoporous structures.

Table S1 summarizes the XPS binding energies of the N 1s and C 1s core levels for CN, Cu_{SA}-CN, and Pt_{SA}-CN samples, together with their corresponding binding energy shifts. For the N 1s spectra, all chemical species exhibit positive binding energy shifts after the incorporation of Cu and Pt single atoms. These shifts are attributed to interfacial electron transfer induced by the strong electronic interaction between the atomically dispersed metal sites and the g-C_3N_4 support. Likewise, the C 1s peaks of the Cu_{SA}-CN and Pt_{SA}-CN samples also shift toward higher binding energies compared to pristine CN, further confirming the modulation of the electronic structure caused by the incorporation of metal single atoms.

Table S1. Binding energy shifts of the N 1s and C 1s XPS peaks for CN, Cu_{SA}-CN, and Pt_{SA}-CN samples.

		CN	Cu_{SA}-CN	Pt_{SA}-CN	ΔB.E (eV) Cu_{SA}-CN	ΔB.E (eV) Pt_{SA}-CN
N1s	N-(C)$_3$	401.38	401.58	401.63	0.2	0.25
	C-NH	400.38	400.53	400.58	0.15	0.20
	C-N=C	398.68	398.78	398.88	0.1	0.20
	C-NH$_2$	399.43	399.58	399.68	0.15	0.25
C1s	N-C=N	287.9	288.06	288.11	0.16	0.21
	N-C-O	289.0	289.26	289.11	0.26	0.11

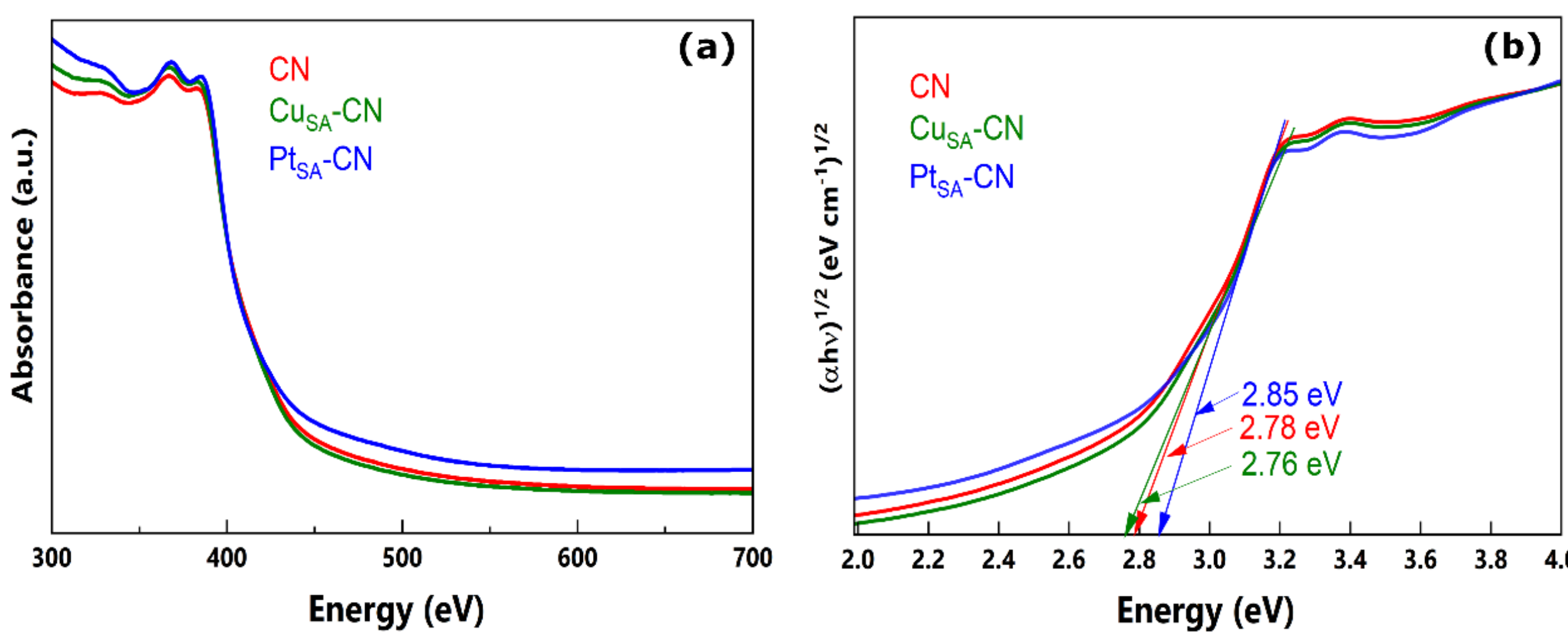

Figure S3. (a) UV-Vis absorption spectra and (b) Tauc plot of pure, Cu_{SA}-CN and Pt_{SA}-CN.

The morphology and dispersion of the metal nanoparticles deposited on bulk g-C_3N_4 were investigated by transmission electron microscopy (TEM), as shown in Figure S4. Both Cu and Pt nanoparticles are distributed over the surface of the g-C_3N_4. The Pt and Cu nanoparticles were deposited onto g-C_3N_4 through a wet chemical impregnation method followed by chemical

reduction. The nominal metal loadings were calculated to be 0.8 wt% for Pt NPs/g-C_3N_4 and 0.5 wt% for Cu NPs/g-C_3N_4, resulting in the formation of nanoscale metallic domains anchored on the support surface.

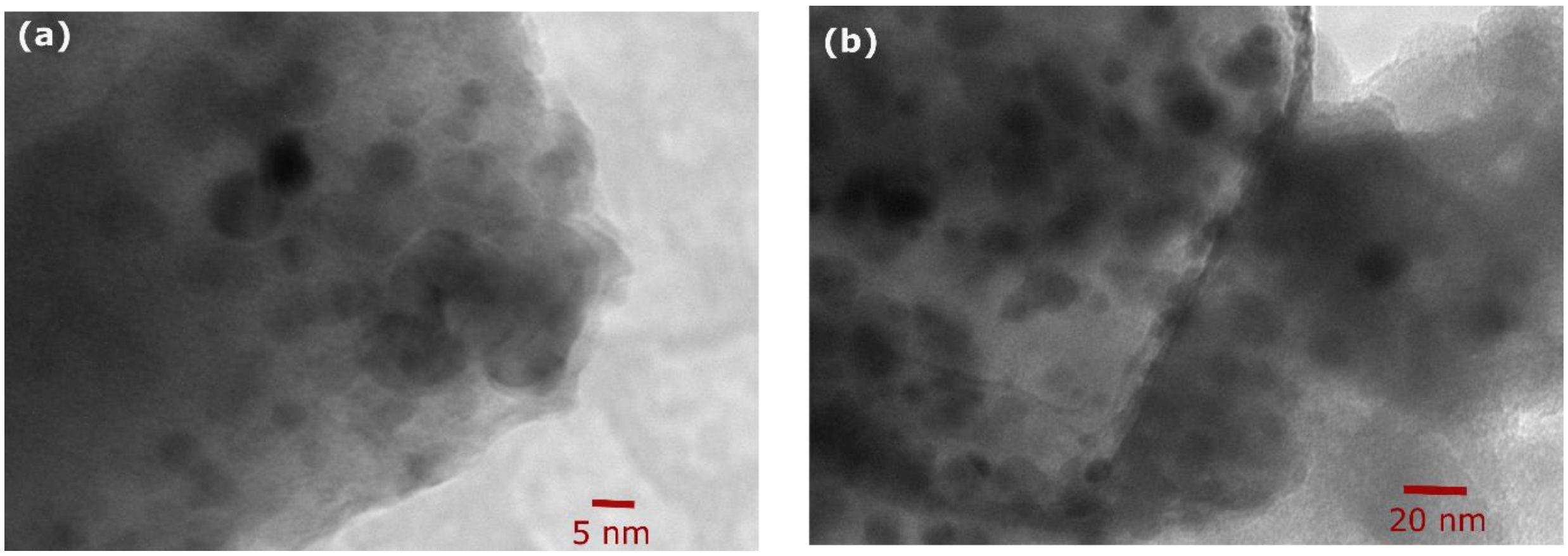

Figure S4. TEM images of (a) Cu nanoparticles (Cu NPs) and (b) Pt nanoparticles (Pt NPs) deposited on g-C_3N_4 by wet chemical impregnation followed by chemical reduction.

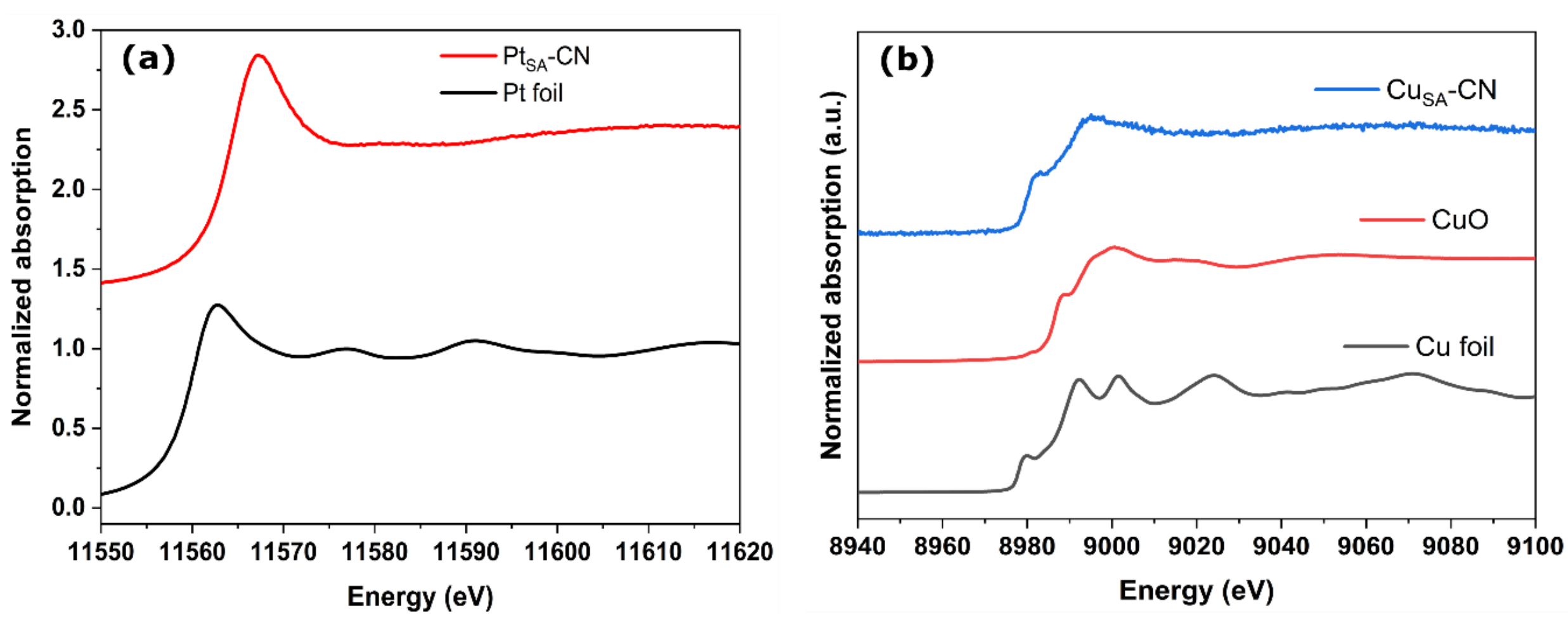

Figure S5. Normalized XANES spectra of (a) Pt_{SA}-CN at the L_3-edge and (b) Cu_{SA}-CN at the K-edge

The photoluminescence (PL) spectra of pristine g-C_3N_4 (CN) and metal-modified samples (Cu and Pt as nanoparticles (NPs) or single atoms (SA)) exhibit a broad emission band centered at ~450–470 nm, which is characteristic of band-to-band recombination in graphitic carbon nitride.[2] The PL spectra of Pt_{SA}-CN and Cu_{SA}-CN samples are compared in Figure S6a, where pristine g-C_3N_4 shows the highest emission intensity, indicating a high recombination rate of photogenerated charge carriers. While the Pt and Cu single atoms deposited on pristine g-C_3N_4 (Pt_{SA}-CN and Cu_{SA}-CN samples) exhibit a significant quenching of the PL intensity. Similarly, the PL spectra of the Cu and Pt NPs deposited on the pristine g-C_3N_4 are shown in Figure S6b, where a significant quenching of the PL intensity is observed, with the most pronounced effect for Cu@gC_3N_4, followed by Pt@gC_3N_4. This behavior suggests that both Cu and Pt act as electron sinks, promoting charge separation by facilitating electron transfer from the g-C_3N_4 matrix. [3] Notably, the stronger quenching observed for nanoparticle-based systems compared to single-atom counterparts indicates a higher density of available active sites for charge trapping and transfer. Additionally, the preservation of the spectral shape across all samples, particularly the long emission tail extending toward 650 nm, indicates that the fundamental electronic structure of g-C_3N_4 is retained, while non-radiative recombination pathways are enhanced. These results are consistent with improved charge separation efficiency, which is directly correlated with enhanced photocatalytic hydrogen evolution activity.

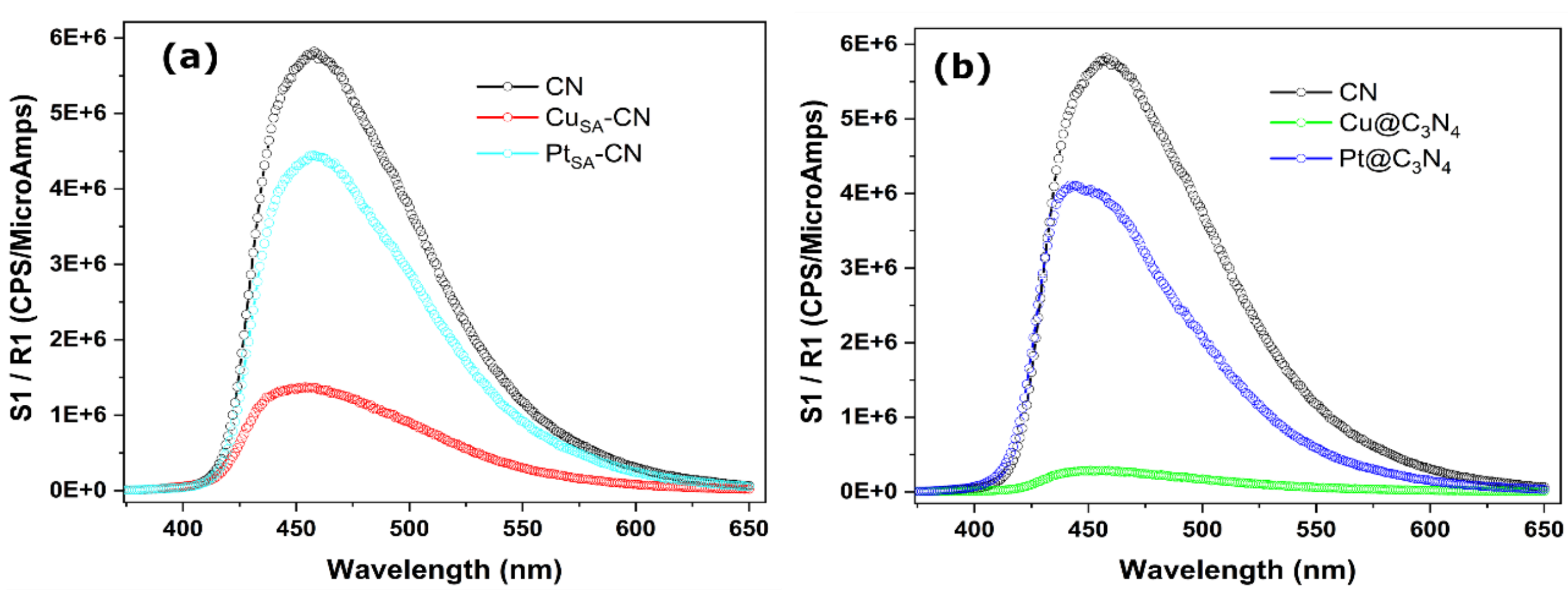

Figure S6. PL analysis of (a) Pt and Cu single atoms and (b) Pt and Cu NPs loaded on g-C_3N_4.

References

(1) Arkhurst, B.; Guo, R.; Gunawan, D.; Oppong-Antwi, L.; Ashong, A. N.; Fan, X.; Rokh, G. B.; Chan, S. L. I. Scalable Fabrication of High Surface Area G-C3N4 Nanotubes for Efficient Photocatalytic Hydrogen Production. *Int. J. Hydrogen Energy* **2024**, *87* (19), 321–331. https://doi.org/10.1016/j.ijhydene.2024.09.006.

(2) Dong, F.; Li, Y.; Wang, Z.; Ho, W. K. Enhanced Visible Light Photocatalytic Activity and Oxidation Ability of Porous Graphene-like g-C3N4 Nanosheets via Thermal Exfoliation. *Appl. Surf. Sci.* **2015**, *358*, 393–403. https://doi.org/10.1016/J.APSUSC.2015.04.034.

(3) Moussa, M.; Hamrouni, A.; Lazaar, N.; Ferhi, M.; Chérif, I.; Lachheb, H.; Silva, C. G.; Sampaio, M. J.; Faria, J. L. Pt-Doped g-C3N4 Photocatalyst for Simultaneous Hydrogen Production and Value-Added Chemical Synthesis under Visible Light. *Photochemical & Photobiological Sciences 2025 24:2* **2025**, *24* (2), 247–259. https://doi.org/10.1007/S43630-025-00683-1.
